# Examining Senior Drivers’ Attitudes Toward Advanced Driver Assistance Systems After Naturalistic Exposure

**DOI:** 10.1093/geroni/igaa017

**Published:** 2020-06-18

**Authors:** Dan Liang, Nathan Lau, Stephanie A Baker, Jonathan F Antin

**Affiliations:** 1 Grado Department of Industrial and Systems Engineering, Virginia Tech, Blacksburg; 2 Virginia Tech Transportation Institute, Blacksburg

**Keywords:** ADAS, Focus group, Naturalistic driving study, Technology acceptance, Topic modeling

## Abstract

**Background and Objectives:**

The increasing number of senior drivers may introduce new road risks due to age-related declines in physical and cognitive abilities. Advanced driver assistance systems (ADAS) have been proposed as solutions to minimize age-related declines, thereby increasing both senior safety and mobility. This study examined factors that influence seniors’ attitudes toward adopting ADAS after significant exposure to the technology in naturalistic settings.

**Research Design and Methods:**

This study recruited 18 senior drivers aged 70–79 to drive vehicles equipped with ADAS for 6 weeks in their own environments. Afterward, each participant was enrolled in 1 of the 3 focus group sessions to discuss their changes in attitude toward ADAS based on their driving experiences. We applied structural topic modeling (STM) on the focus group transcripts to reveal key topics deemed important to seniors.

**Results:**

STM revealed 5 topics of importance for seniors. In order of prevalence, these were (i) safety, (ii) confidence concerning ADAS, (iii) ADAS functionality, (iv) user interface/usability, and (v) non-ADAS–related features. Based on topics and associated keywords, seniors perceived safety improvement with ADAS but expressed concerns about its limitations in coping with adverse driving conditions. Experience and training were suggested for improving seniors’ confidence in ADAS. Blind spot alert and adaptive cruise control received the most discussion regarding perceived safety and comfort.

**Discussion and Implications:**

This study indicated that promoting road safety for senior drivers through ADAS is feasible. Acceptance and appropriate use of ADAS may be supported through intuitive and senior-friendly user interfaces, in-depth training programs, and owner’s manuals specifically designed and tested for senior drivers.

**Translational Significance:** After driving in vehicles equipped with Advanced Driver Assistance Systems, seniors indicated their appreciation for blind spot alert and adaptive cruise control for their safety and convenience benefits. Seniors expressed concerns about usability and robustness of some advanced features, recommending that manufacturers and retailers provide in-depth training with effective documentation for understanding the limitations of these features and to promote usage by designing more intuitive and senior-friendly user interfaces.

The U.S. Census Bureau (2018) predicts that the population of seniors aged 65 and older will reach 94.7 million by 2060, making up about a quarter of the U.S. population. The number of senior drivers is projected to increase from 42 million (Federal Highway Administration, 2016) to more than 60 million by 2030 (AAA Exchange, 2017). More senior drivers on the road could raise road safety concerns. Though not all senior drivers are at high risk of traffic accidents, they do have a higher likelihood of being involved in fatal crashes due to increased fragility (Insurance Institute for Highway Safety, 2013). While seniors may have years of driving experience, they often show mild impairments that could impact driving safety. Examples include poor vision (e.g., Anstey, Wood, Lord, & Walker, 2005; Attebo, Mitchell, & Smith, 1996), declining memory performance (e.g., Perlmutter & Nyquist, 1990; Rabbitt, 1965; West, Crook, & Barron, 1992), divided attention-related failures (e.g., Ponds, Brouwer, & Van Wolffelaar, 1988; Salthouse, Mitchell, Skovronek, & Babcock, 1989), and slower reaction time (Marottoli & Drickamer, 1993).

Emerging advanced driver assistance system (ADAS) technologies have the potential to improve safety and mobility for senior drivers by compensating for some of these milder impairments. ADAS is a general term that includes a broad array of features. In the context of this article, the term ADAS refers to features at the lower three SAE International levels (Levels 1–3) of driving automation (SAE International, 2014), excluding those only in highly automated and driverless vehicles. ADAS can sense and provide important driving information, such as vehicles in the blind spot with blind spot alert (BSA), and help control the vehicle in very specific conditions, such car following with adaptive cruise control (ACC). The potential safety benefits can be substantial for seniors experiencing mild cognitive or physical declines. For example, seniors with neck rotation difficulties may find a BSA particularly helpful. Researchers have been optimistically anticipating that ADAS will alleviate age-related safety decrements commonly manifested in driving (e.g., Band & Perel, 2007; Caird, 2004; Davidse, 2006; Eby et al., 2016; Meyer, 2014).

Attaining anticipated ADAS benefits is dependent on drivers’ acceptance of and adaptation to the capabilities of new technologies and thus a new way of driving. Relative to other age groups, seniors seem more reluctant (Caird, 2004), sometimes even resistant, to adopt innovative technologies (Tacken, Marcellini, Mollenkopf, Ruoppila, & Szeman, 2005). Trust in technology also seems to decrease with age (Ho, Kiff, Plocher, & Haigh, 2005), and learning new skills and changing well-established routines become more difficult with age (Craik & Jacoby, 1996). However, seniors have indicated that they may adapt to advanced technologies as readily as younger groups if provided with sufficient training and exposure opportunities (Owens, Antin, Doerzaph, & Willis, 2015). Thus, policy makers and manufacturers must understand the factors influencing senior drivers’ attitudes toward and adaptation to using ADAS to alleviate aging-related traffic risks.

Most studies on seniors’ attitudes toward ADAS have employed surveys on a large sample of drivers from all age groups and sometimes have held focus groups comprising various user populations. Seniors generally have significantly lower technology utilization and acceptance rates than younger and middle-aged users (Czaja & Sharit, 1998), which is reflected in their lower inclination to use ADAS (Bansal, Kockelman, & Singh, 2016; Munich, 2017). Through a focus group study about a lane departure warning system, Regan, Mitsopoulos, Haworth, and Young (2002) found that seniors were not willing to pay as much for the feature as young adults were. Other studies also found that, in general, seniors appeared less willing to pay for ADAS, highly automated, or driverless technologies (Abraham et al., 2016; Bansal & Kockelman, 2018; Bansal et al., 2016; Payre, Cestac, & Delhomme, 2014; J.D. Power, 2012). These survey studies illustrate differences in attitudes toward ADAS between seniors and drivers in other age groups.

In comparison to other age groups, seniors may be less willing to accept ADAS, but studies focused specifically on seniors reveal substantial nuances in their perception of advanced vehicle technologies. In terms of awareness, Davern, Spiteri, and Glivar (2015) conducted a two-phase study, in which eight 45-min in-depth interviews were conducted with eight senior drivers, and then an online survey was administered to 1,070 seniors. Participants were required to be over the age of 60 for both phases. Findings revealed that seniors generally had limited knowledge of the latest vehicle technologies and ranked “features/technologies within the car space” to be the most important factor in their perceptions of car safety.

The Hartford Center for Mature Market Excellence and the MIT AgeLab conducted a series of nationwide surveys on drivers more than 50 years of age to assess ADAS and driverless technology acceptance, preference, and system learning. Collectively, the main findings of the studies were that (i) most seniors were willing to purchase a car with at least one ADAS (The Hartford, 2015, 2017), citing safety as their primary reason (The Hartford, 2012a, 2012b, 2015, 2016); (ii) seniors were most willing to adopt BSA (in-vehicle alerts warning of objects in blind spots; The Hartford, 2012a, 2015, 2016); and (iii) seniors first relied on an owner’s manual, second on trial and error, and finally on car dealers to learn about the ADAS installed in their own cars (The Hartford, 2012a). One survey highlighted seniors’ preference for well-designed driver education programs, such as workshops, online tutorials, and hands-on learning with an instructor driving, to be provided by trusted organizations or dealers (The Hartford, 2017).

Besides surveys, the literature includes interview and focus group studies. An early focus group study investigated the expectations of British senior drivers (aged 52–79) on emergency signaling, navigation systems, fatigue monitoring, and forward collision avoidance systems (Sixsmith, 1990). Seniors expressed skepticism about the warning features, which could take their attention away from driving, but they were receptive to systems providing real-time information on road conditions. Oxley (1996) investigated reactions of seniors aged more than 65 to in-vehicle navigation, rear collision warning, an emergency notification (“Mayday”) system, and night vision enhancement after they had experience with the systems under examination. Seniors found that navigation systems were distracting but that night vision enhancement was highly acceptable. Generally, participants showed a high willingness to adopt and purchase ADAS, provided that the benefits were perceived to be real and the design was perceived to suit their needs.

Recently, Marshall, Chrysler, and Smith (2014) conducted a focus group study with 51 participants aged 55–75 to rate their acceptance of four ADAS categories: enhancement, alert, vehicle control, and fully automated/connected vehicle systems. The highest acceptance score was for systems providing alerts as necessary while allowing drivers to remain in control (e.g., forward collision warning systems). Vehicle control systems (e.g., ACC systems) received a relatively low acceptance rating due to issues of trust, distraction, and overreliance.

Researchers conducted semistructured interviews with 32 drivers aged 60–80 to explore their experience with and barriers to using ADAS (Trübswetter & Bengler, 2013). Results revealed that the most common reason that seniors avoided using ADAS was that they perceived little in the way of benefits, followed by the beliefs that ADAS provided limited functionality, were high in cost, and were untrustworthy. Gish, Vrkljan, Grenier, and Van Miltenburg (2017) interviewed 35 seniors who had at least two ADAS in their vehicles about their perception of and motivation for using the technology. The interviews revealed that ADAS were perceived to counteract age-related declines but were not the motivating factor in purchasing decisions. Hence, participants who were exposed to ADAS valued the safety benefits as well as the convenience and comfort.

In short, while the literature contains surveys, interviews, and focus group studies on seniors’ perception, acceptance, and preferences in regard to ADAS, these studies do not carefully consider or control for exposure and experience with ADAS. Thus, how seniors perceive, accept, and actually use ADAS requires further investigation, especially as these technologies are rapidly proliferating into the existing vehicle fleet. The objective of our study is to examine the underlying factors affecting changes in senior drivers’ perceptions and attitudes toward ADAS after substantial driving exposure.

## Research Design and Methods

### Data Collection

Eighteen seniors (nine men and nine women) were recruited to participate in a driving study in the New River Valley area of Southwest Virginia. Participants were recruited from a list of individuals who had previously indicated an interest in serving as a participant in Virginia Tech Transportation Institute research efforts. Eligibility criteria included age (70–79), ADAS experience (never owned an ADAS-equipped vehicle), driving regularity (at least 2 days per week), a valid driver’s license, and insurance coverage. We considered the needs and preferences of the individual when assigning the vehicles. For example, if a participant preferred a Volvo based on current or prior ownership, we tried our best to allocate a Volvo to that individual. If there were no special needs or preferences, we randomly assigned study vehicle models to individual participants. The study began with an intake session during which potential participants showed their driver’s license and proof of liability insurance. After providing informed consent, participants were given questionnaires to collect demographics, driving habits, and history, as well as their pre-exposure attitudes toward ADAS. Each participant was then assigned to one of the four vehicle models (2017 Audi Q7, 2016 Mercedes E350, 2016 Volvo XC90, 2015 Infiniti Q90). Each of these vehicles was equipped with at least the following four ADAS: BSA, lane alert (LA), lane keeping assist (LKA), and ACC. The Supplementary Material section includes further details about the implementation of these four ADAS for each of the four vehicle models used in this study.

After being assigned to a study vehicle, participants received a three-part training session. The first part was performed in the parked vehicle while an experimenter explained the basic vehicle features (e.g., windshield wipers, gear shift selector, etc.) and how the four ADAS functioned. The second part was an on-road drive in which the experimenter drove the vehicle and demonstrated how to use the four ADAS common to all the vehicles used in this study. The experimenter also briefly mentioned the scenarios or environments in which the participants should try to avoid using ADAS given the technology limitations. In the third part, the participant drove the vehicle, using or experiencing each of the four common ADAS, based on the experimenter’s verbal guidance. The on-road drive was designed to provide training on the proper use of ADAS under practical conditions on highways in the New River Valley area. The entire training session lasted 1.5–3 hr.

After training, participants were asked to drive the study vehicle as they normally would for a 6-week period. Weekly phone surveys were conducted to collect data on participants’ attitudes about and usage of the vehicles and each ADAS. Upon return of the vehicles, the same questionnaire used at the beginning of the study was readministered to collect participants’ postexposure attitudes toward the ADAS.

Within 2 weeks of returning the study vehicle, each participant took part in a 90-min focus group session. Three focus groups were conducted, each of which included six participants. During the focus groups, participants shared their opinions about and perspectives on the ADAS through the following series of specific guiding questions:

(1) What one word describes how you felt about the advanced features in your vehicle when you began the study?
(2) What one word describes how you felt about the advanced features in your vehicle at the end of the study?
(3) What caused your feelings change or remain the same?
(4) What would make you feel more comfortable with these features?
(5) What is one thing you liked best about these features?
(6) What is one thing you liked least about the features?
(7) Suppose a friend is considering purchasing a car with these features and they ask you if you think if they improve driving safety or not. What would you say?

The guide questions were all open-ended and designed to address the main research question of seniors’ attitudes toward the ADAS controlling for exposure (i.e., seniors’ attitudes toward ADAS, how they liked or disliked certain systems, and their perceptions regarding the safety benefits of the ADAS).

A skilled moderator facilitated the focus group sessions, guiding discussions to ensure responses were being provided by all participants and asking probing questions as necessary. Two other researchers also attended each session to take notes and monitor the recording equipment. Three researchers transcribed the focus group recordings verbatim. The study protocol was approved by the Virginia Tech Institutional Review Board.

### Data Analysis

Topic modeling is an unsupervised text analysis method that recognizes, classifies, and extracts information by clustering words frequently appearing together across a collection of documents to identify different and prevalent topics (Blei, 2012). This computer-assisted text analytical method has multiple advantages over the standard practice of using a human subjective coding procedure in focus group analysis. Benefits of topic modeling included avoiding the issue of observer dependency bias, greater speed in processing large volumes of text, and consistent treatment of all documents (Grimmer & King, 2011; Hillard, Purpura, & Wilkerson, 2008; Lowe & Benoit, 2013; Quinn, Monroe, Colaresi, Crespin, & Radev, 2010). For our data set, the collection of documents consisted of the transcripts from all three focus groups. Each document was composed of text transcriptions of the discussion (i.e., all verbal exchanges from all participants and the moderator) on one guiding question in one of the three focus groups. Thus, the complete collection comprises 24 documents, given that there were seven guiding questions and an open discussion section in each of the three focus group sessions.

Based on a latent Dirichlet allocation algorithm (Blei, 2012; Blei, Ng, & Jordan, 2003), structural topic modeling (STM; Roberts, Stewart, Tingley, & Airoldi, 2013; Roberts, Stewart, Tingley, Lucas, et al., 2014) was selected to obtain the following results: (i) a set of topics, (ii) a set of keywords to represent each topic, (iii) prevalence/expected proportion of each topic in the collection, (iv) per-document-per-topic (gamma) probabilities. All data processing and analysis was conducted with an STM package in R (Roberts, Stewart, & Tingley, 2014).

### Data Preprocessing

In order to apply STM, the transcripts were preprocessed as follows:

(1) Remove introduction and greetings at the beginning of each focus group as well as the introductory sentences under each guide question.
(2) Convert different word phrases representing the same ADAS feature to one acronym such as “ACC” for “adaptive cruise control,” “ACC,” and “cruise control.” [In our analysis, we converted “cruise control” to ACC only when participants were discussing ACC specifically. We converted three instances of “cruise control” to CC when participants discussed traditional cruise control.] BSA was used for blind spot alert, LKA for lane keeping assist, and LA for lane alert.
(3) Remove all common stop words, such as “a,” “the,” and “we,” which have limited semantic value.
(4) Remove customized stop words, such as “car,” “vehicle,” and “driving.”
(5) Consolidate words with different tenses or forms to their word stem, such as, “experienc” for “experiencing,” “experienced,” and “experience.”

### Number of Topics

**Figure 1.**
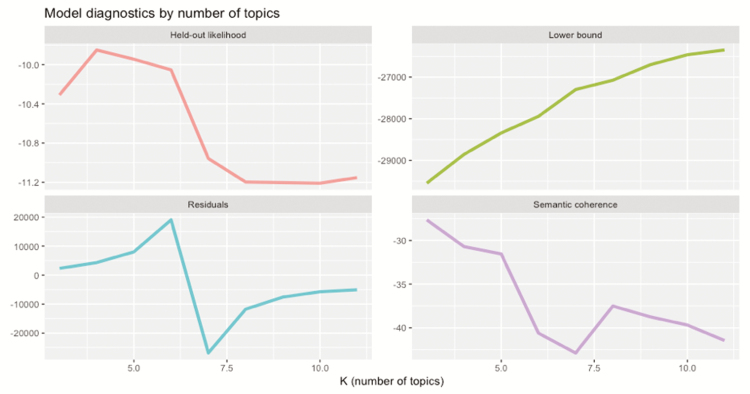
Held-out likelihood, lower bound, residuals, and semantic coherence scores for models with 3–10 topics.

The number of topics must also be preset by the analyst in order to apply STM. Typically, the number of topics is determined by exploring the data set to compute four metrics: held-out likelihood, sematic coherence, residual, and lower bound. To find the model that produces the most semantically coherent and distinct topics, the four metrics were computed for a range of 3–10 topics, based on recommendations by Benoit (2018). The five-topic model was selected for its relatively high semantic coherence and held-out likelihood while maintaining a relatively low residual and lower bound (Figure 1; Blei et al., 2003). Semantic coherence measures the co-occurrence of words within the documents to ensure selected keywords belong to a single concept, thus preserving the interpretability or quality of the topic (Mimno, Wallach, Talley, Leenders, & McCallum, 2011). Held-out likelihood estimates the probability of keywords appearing in documents (excluded or held-out of the training) to indicate the generalization capability of the topic model (Wallach, Murray, Salakuhutdinov, & Mimno, 2009).

## Results

### Topic and Prevalence

Table 1 presents the prevalence and the most representative words based on *Prob* and *FREX* metrics for each topic. *Prob* is the probability of occurrence of a term within a given topic, whereas *FREX* (“FRequency and EXclusivity”) is calculated based on the frequency of the word and its degree of exclusivity to a particular topic (Airoldi & Bischof, 2016; Bischof & Airoldi, 2012). Researchers assigned a label to each topic based on inspection of its content. The first four topics pertain to the primary interests of this study, while the responses associated with Topic 5 mainly contain vehicle components that are not related to ADAS. We display the content for all five topics in the following section, but there is no further discussion on Topic 5.

**Table 1.** Topic Label, Keywords, and Topic Proportion

No. topic label	Metric	Keywords	Topic proportion
1. Safety	Prob FREX	safeti*, control, improv*, lane, chang*, set, bsa improv*, headlight, rain, safeti*, friend, bright, construct*	0.25
2. Confidence concerning ADAS	Prob	confid*, lane, control, acc, chang*, experi*, manual	0.22
	FREX	pleas*, confid*, equip*, lane, center, convinc*, stai*	
3. ADAS functionality	Prob	acc, seat, bsa, set, light, slow, comfort*	0.22
	FREX	seat, head, bsa, spot, advanc*, miss, slow	
4. User interface/usability	Prob	heat, figur*, button, trainer, confus*, learn, nervou*	0.17
	FREX	intuit*, heat, train, press, sound, figur*, confus*	
5. Non-ADAS–related features	Prob	wheel, steer*, light, stop, signal, brake, acc*	0.14
	FREX	stop, hang, signal, placement, son, technologi*, rear	

*Notes.* ADAS = advanced driver assistance systems. Asterisks indicate stemming. For example, the term “confid*” refers to both “confidence” and “confident.” Translations are best approximations based on readings of representative quotes.

### Per-Document-Per-Topic Probabilities

Table 2 presents the per-document-per-topic (gamma) probabilities for the three focus groups (Silge & Robinson, 2017). The gamma probabilities indicate how much individual documents contribute to each topic. All three focus groups discussed the three most prevalent topics (i.e., safety, confidence concerning ADAS, and ADAS functionality). However, focus Group 1 had virtually no discussion on Topic 5 (non-ADAS–related features), nor did focus Group 3 have any substantive discussion on Topic 4 (user interface/usability). However, in general, the three focus groups shared the same discussion topics, especially those that were most prevalent.

**Table 2.** Per-Document-Per-Topic (Gamma) Probabilities

Group	Document no. (section)	Topic 1	Topic 2	Topic 3	Topic 4	Topic 5
Group 1	1. Pre attitude	0.0019	0.0073	0.0018	**0.9869**	0.0021
	2. Post attitude	0.0004	**0.9976**	0.0008	0.0005	0.0007
	3. What makes change	0.0021	**0.9870**	0.0041	0.0028	0.0040
	4. Perception	0.0007	0.0009	0.0007	**0.9969**	0.0007
	5. Like most	0.0032	0.0042	**0.9900**	0.0012	0.0014
	6. Like least	**0.9951**	0.0012	0.0014	0.0012	0.0011
	7. Safety	**0.9954**	0.0009	0.0019	0.0009	0.0009
	8. Open discussion	**0.9965**	0.0008	0.0011	0.0007	0.0009
Group 2	9. Pre attitude	0.0016	0.0020	0.0016	**0.9931**	0.0016
	10. Post attitude	0.0017	**0.9923**	0.0026	0.0013	0.0021
	11. What makes change	0.0062	**0.5796**	0.0049	0.0046	**0.4047**
	12. Perception	0.0008	0.0008	0.0007	**0.9970**	0.0007
	13. Like most	0.0014	0.0019	**0.9950**	0.0007	0.0009
	14. Like least	**0.9951**	0.0013	0.0013	0.0010	0.0013
	15. Safety	**0.9973**	0.0006	0.0009	0.0006	0.0006
	16. Open discussion	0.0011	0.0015	**0.9958**	0.0009	0.0008
Group 3	17. Pre attitude	0.0009	0.0016	0.0009	0.0008	**0.9960**
	18. Post attitude	0.0011	**0.7097**	0.2842	0.0009	0.0041
	19. What makes change	0.0015	**0.9906**	0.0049	0.0017	0.0033
	20. Perception	0.0008	0.0013	0.0008	0.0006	**0.9966**
	21. Like most	0.0012	0.0017	**0.9955**	0.0007	0.0009
	22. Like least	0.0012	0.0017	**0.9952**	0.0008	0.0011
	23. Safety	**0.9958**	0.0012	0.0012	0.0007	0.0011
	24. Open discussion	0.0016	0.0021	0.0016	0.0011	**0.9937**

*Notes.* ADAS = advanced driver assistance systems. The numbers in bold highlight the major contributions of individual documents to a topic.

*Topic 1: Safety* ranked as the most prevalent topic (0.25). These entries emphasized two aspects of safety-related discussions. On the one hand, seniors appreciated the safety benefits associated with using certain aspects of the ADAS–BSA and adaptive headlights, specifically. On the other hand, limited capability and false alerts when driving through a construction area or in inclement weather conditions resulted in safety-related concerns. Representative quotes regarding this topic are outlined in Table 3 (Quotes 1–6).

**Table 3.** Representative Responses by Induced Topics

Topic	Representative responses
1. Safety	1. It improves safety as long as you’re still in control. 2. Yes, improves safety. And, you will love the blind spot alert. 3. There were all kinds of things I liked about the car, not the car itself, but they weren’t in these safety features [ADAS] we were supposed to be concentrating on. I liked not having to turn on my headlights. They were on all the time and they did adjust really well to a brightness or any time when I was driving in the bright daylight and then it started to rain and I noticed that they headlights went brighter. 4. I LOVED and it is not one of these, the fact that if you’re driving the Volvo at night, maybe yours didn’t, it adjusts the lights too. I think that’s fantastic. You know, if you’re in the dark and you’re going around a curve, it will brighten it for you. Yeah, that was really neat. 5. The lane changing. One of the things about the lane changing at least because I was on 460 all the time, is particularly in construction areas. You know when the roads are divided by the white lines and part of it’s the road and part of it is the construction. It doesn’t seem to quite know it’s vibrating in the wrong place. 6. Uh, if, if the wind is blowing, it doesn’t work. If it’s raining, it told me it wasn’t working, you know, all these situations and, and at night and I’m like well I’m a senior driver and I need help in these situations more than when I, when it works.
2. Confidence concerning ADAS	7. By using the system, I became more confident and also by testing various things. 8. I felt confident with the systems. I had done enough to know that within its limitations it did what it was supposed to do. And then I also felt confident that I knew that there was some parts of it that I couldn’t, I couldn’t rely on in certain situations. 9. Experiencing the features. At first I was wondering if they were going to be effective, if I could actually use them to my benefit. And after experiencing them, you know, it convinced me that it was a good thing. 10. Confident. Oh, well, I, um, I used the car as much as I possibly could. So I think practice, you know, made the awkwardness go away. Wasn’t long before I knew where each little control was without having to look for it. You know, I could feel where it was and everything. So I also felt confident about the fact that I gave it a fair trial. That I tried every little thing I could think of to see how it behaved and how it worked and I felt good about that. 11. Just experience and time to play with it. Trying it, you know like trying to see if I could get onto 460 and put on the lane control, see if I could drive out to Lowes without using my hands, did not work. 12. Well, the owner’s manual is four inches thick and it’s not well written. In the sense that where you go to find, how do I do this? It’ll say push the something button, but then it doesn’t tell you where the something button is and I just found it really hard to learn. 13. Well, from reading the manual and trying things, you know, figuring out how it worked by the time I brought the car back, I knew where the functions were and how to use them and so forth. 14. In the manual I have, it outlined the limitations very clearly. So all of that made me feel how much, you know, really better about the whole system.
3. ADAS functionality	15. I like blind spot also, even though it was there I was in the habit of turning my head away, but it alerts me to turn my head to double check that there is nothing there. 16. BSA is an enhanced safety for me ... as the arthritis sets in more and more in my neck and spine, turning around is not easy. 17. After I brought the car back and I was driving my car again, then I missed some of the features. I, you know, I catch myself looking for the light [BSA] that blinks and the light wasn’t there. 18. I’m not somebody who likes cruise control, but I fell in love with the adaptive cruise control. 19. The ability to [drive] along with that adaptive cruise control, the ability to set the comfortable distance for yourself because people drive differently. 20. I felt safer. Cause our mind wanders when we’re driving, and it did happen to me twice, I made notes, that I wasn’t paying attention, and it all of a sudden, I said, why am I slowing down? and there was a car in front of me! 21. Well, let’s say, the ACC, I use ACC all the time if I’m on the interstate or open road, but whenever that thing kicks in and slowed me down, because the car in front of me, I hit the brake knock it out and can sneak on up there and get around.
4. User interface/usability	22. I was just nervous about using it and not being able to... At first it was hard to find some. For example, the ACC and the signal light switches are real close together and, if you’re not familiar with them, you hit the wrong things. Well I’d be wanting to hit the signal light and I’d hit the cruise and speed up you know, so I was a little nervous about using them. And to this day I haven’t figured out how to set the radio.
	23. I think the controls could be … I didn’t find them very intuitive as far as their location. And so I think that the controls should be relocated, maybe to a panel where they’re isolated these special safety features all in one panel maybe instead of trying to remember is it on this stalk or that stalk or this little button under here. I just think it could be more intuitive as far as the controls are concerned. 24. I couldn’t figure out how to turn the heat off and get the AC on. 25. You just pushed a button to turn on the features but you couldn’t decide which features to be on. 26. I had to read the book [manual] to figure out what where that was, now it was under climate I found, but I didn’t know that right away. 27. So some in-depth training on how to turn things on, when to use them, when you want them on … .
5. Non-ADAS–related features	28. Now, the seat did not move but it was the steering wheel and I really liked that. 29. It wasn’t comfort with the features, it was comfort with the seats. 30. What I mentioned before, the, the, the driver’s seat, which is way back. You can’t, can’t even put your foot on the accelerator when you get in the car and then you have to start the car and, and it moves the seat up about a foot and a half and it was just annoying. It’s a, you do not need that. 31. If you have a handicap tag, there’s no place to hang your handicap tag.

*Notes.* ADAS = advanced driver assistance systems. The selected quotes are from the representative answers by topic, based on a qualitative assessment of their responsibility.

*Topic 2: Confidence concerning ADAS* ranked as the second most prevalent topic (0.22). This topic expresses two aspects of confidence. First is confidence in ADAS functioning effectively on the road. Practice with ADAS and seeing the systems working well changed participants’ attitudes toward ADAS from apprehensive to confident. Second is the self-confidence in using ADAS appropriately. Experience and reading the vehicle manual appear to be the two methods that promote seniors’ self-confidence in using ADAS. Though some had negative comments on readability and a lack of sufficient detail, participants agreed that the vehicle owner’s manual plays an important role in building both aspects of confidence noted above during the early adoption period. Participants gained familiarity with and learned some of the limitations of the ADAS from the manual. Representative quotes regarding this topic are outlined in Table 3 (Quotes 7–14).

*Topic 3: ADAS functionality* tied for second in topic prevalence (0.22), highlights the particular ADAS technologies that made the biggest impression on seniors—BSA and ACC. This topic overlaps with Topic 1 regarding perceived safety benefits. BSA helps seniors with neck problems manage the blind spot without turning their heads, and thus is specifically associated with helping counter the effects of aging. Participants perceived BSA to counteract limitations in neck flexibility and rotation. ACC was also frequently discussed. Some participants showed appreciation for the capability of automatically keeping the vehicle at a comfortable distance from a leading vehicle. However, other participants disliked being slowed down by the ACC and expressed sometimes preferring to disengage the technology and pass the slower leading vehicle. Representative quotes regarding this topic are outlined in Table 3 (Quotes 15–21).

*Topic 4: Usability/user interface* ranked fourth in topic prevalence (0.17) concerns usability issues. Some participants were confused about the location of the appropriate ADAS controls or about how to operate the function that they intended to use. The user interfaces were not intuitive to them. Accompanying complaints about usability were requests for in-depth training, going through how to turn on/off all functions, including those not related to ADAS. Representative quotes regarding this topic are outlined in Table 3 (Quotes 22–27).

*Topic 5: Non-ADAS–related features*, the least prevalent topic (0.14), surrounded issues that were not directly related to participants’ ADAS experience. Participants discussed car interior designs and components, such as car seats, steering wheel, handicap tag hanger, and various luxury features. Representative quotes regarding this topic are outlined in Table 3 (Quotes 28–31).

## Discussion and Implications

This is the first naturalistic driving study investigating seniors’ attitudes toward and experience in using ADAS based on significant driving exposure. Focus groups were conducted after participation in the driving portion of the study. STM performed on the focus group transcripts revealed five prevailing topics or factors of importance to participants.

These five topics, in order of prevalence as revealed by the STM, are (i) safety, (ii) confidence concerning ADAS, (iii) ADAS functionality, (iv) user interface/usability, and (v) non-ADAS–related features.

The safety implications of ADAS were foremost in the minds of study participants, which is consistent with the finding that seniors consider the safety of a vehicle as a primary criterion in their purchase decisions (Davern et al., 2015). Similarly, in a survey asking about purchasing a self-driving vehicle, results showed that mature drivers (aged 50 and older) would consider such a purchase if the self-driving vehicle was proven to be as safe as if the participants were driving the vehicle themselves (The Hartford, 2016). Participant impressions in this study were mixed in that ADAS elicited both positive feelings about safety improvements as well as concerns regarding false alerts and a limited range of effective operations. Terms such as “safety” and “improved” frequently co-occurred in the focus group sessions, indicating that participants believed ADAS brought safety improvements. Similar positive findings of the perceived ADAS safety benefits can be found in previous studies (Davern et al., 2015; Gish et al., 2017; The Hartford, 2015). However, the false alerts that occurred during particular situations (e.g., construction zones) were a concern to participants. Concerns about ADAS are also apparent in the literature, particularly regarding false BSA alerts and LKA and ACC malfunctions in certain driving conditions (Strand, Nilsson, Karlsson, & Nilsson, 2011). In other words, many current LKA and ACC systems are not sufficiently robust for the full range of road conditions, making training an important remedial solution to support drivers in learning about appropriate use of ADAS as well as their inherent limitations.

Seniors indicated that both confidence in the ADAS as well as their self-confidence in using them grew along with their knowledge derived from driving experience and reading the owner’s manual. This topic mirrored the findings by comparing questionnaires collected pre- and post-6-weeks’ exposure to ADAS. Participants reported positive attitude changes toward ADAS, especially in regard to familiarity, lower concern about false alerts, and trust in the effectiveness of the systems regarding safety (Liang et al., 2019). The focus group discussion adds to our finding that the positive attitude change in the survey results can be attributed to the driving experience as well as reading the manual. The Hartford (2012a, 2012b) also found similar results on increased confidence in driving with ADAS and seniors’ preferred learning methods. Similarly, Koustanaï, Cavallo, Delhomme, and Mas (2012) also found that training and experience were essential for drivers to learn about capabilities, benefits, and limitations of a forward collision warning system. Interaction with and first-hand demonstration of ADAS can improve seniors’ perception and understanding of these systems (Davern et al., 2015). Llaneras (2006) indicated that seniors were more likely to learn ACC through the owner’s manual than were other age groups, which is consistent with our findings that seniors used the owner’s manual to gain system knowledge.

Taken together, Topics 1 and 2 indicate that a training program is an essential aspect of promoting sufficient knowledge and calibrated trust that will eventually translate to adoption of an ADAS. However, most current ADAS still need improvement in robust operations across a wide range of driving situations. An imperfect or unreliable system can negatively influence driver trust in the system (Beggiato, Pereira, Petzoldt, & Krems, 2015; Llaneras, 2006). In this study, participants showed signs of ADAS mistrust via expressing concerns about false alerts from LKA in construction zones. On the one hand, competency across driving conditions is essential in helping drivers trust ADAS, as effective operation in a very narrow range of conditions is impractical, if not unsafe, in real-world driving. On the other hand, drivers must also understand and appreciate operational constraints, which exist even in the best technology, in order to avoid mistrust and misuse of ADAS and maximize the system’s expected benefits. Safe cooperation with technology depends on system knowledge, which influences an individual’s attitude (Beller, Heesen, & Vollrath, 2013) and calibrated trust (Chavaillaz, Wastell, & Sauer, 2016; Lee & See, 2004; Sexton & Geffen, 1979). Because seniors have been found to have less ADAS knowledge (Davern et al., 2015), they are more prone to mistrust or use ADAS in inappropriate driving conditions compared with other age groups. As technological improvements of ADAS take time, effective training for seniors must be available and have two key components. First, practical, hands-on experience to provide real-time operational knowledge and familiarity with ADAS is essential for seniors to remain “in-to-the-loop” and avoid automation surprises (Louw & Merat, 2017). Furthermore, familiarity is a good predictor of senior ADAS adoption (Souders, Best, & Charness, 2017). Second, well-written documentation can augment driving experience on real roads given seniors’ greater willingness to read owner’s manuals for ADAS basic knowledge and limitations.

Among the investigated ADAS technologies, BSA and ACC made the biggest impression on seniors in terms of their safety benefits. The positive comments on BSA confirmed the results of multiple survey studies about the willingness to purchase and adopt ADAS (The Hartford, 2012a, 2015, 2016). Seniors appeared most receptive to ADAS that provided alerts only (Marshall et al., 2014); they felt positive, safe, and less stressed with BSA (Braitman, McCartt, Zuby, & Singer, 2010) and were found to value the BSA functionality almost twice as much as younger drivers (Souders et al., 2017). Our participants indicated that turning their heads to check blind spots to be a challenging driving task and found BSA to be helpful in mitigating this difficulty, supporting the findings from Gish and colleagues (2017) about how seniors’ perceptions of ADAS were shaped with respect to their aging bodies.

Interestingly, several participants in our focus group sessions reported that they found themselves still looking for the BSA in their own vehicles, which were not equipped with this feature. BSA has been shown to promote the frequency of mirror checking (Kiefer & Hankey, 2008). Though seemingly promoting safety, this finding leads to an important set of questions not directly addressed in the current study regarding the implications for ADAS trust, usage, and safety when seniors (all drivers, in fact) switch back and forth between ADAS– and non-ADAS–equipped vehicles.

Participants also extensively discussed ACC, reporting that it made them feel more comfortable behind the wheel. Stanton and Young (2005) showed that using ACC was associated with decreased workload and stress in addition to the safety benefits. Vision-related factors during night driving were found to be a significant predictor of seniors’ willingness to use ACC (Souders et al., 2017). Given that night vision declines with age, seniors might find ACC comparatively beneficial and thus be willing to yield control to this technology. However, previous studies also found negative impacts on safety associated with decreased situational awareness (De Winter, Happee, Martens, & Stanton, 2014) and overreliance on ADAS (Rajaonah, Anceaux, & Vienne, 2006). Collectively, from the literature and our study results, senior drivers were found to have positive perceptions of BSA and ACC. This stands in contrast to our participants’ impression of LKA, which was not perceived to perform effectively under many driving and road conditions. Therefore, some ADAS features appear ready for adoption to promote safety when users are provided sufficient training and instruction. Participants identified usability as an area of concern, as they had difficulty locating and operating particular ADAS functions. As seniors tend to have difficulty changing their well-established routines (Craik & Jacoby, 1996), learning to use the latest in-vehicle technologies may require a longer period of adjustment. Prior research has demonstrated that seniors desire more in-depth training on vehicle technology from dealers or other instructors (The Hartford, 2017). Any such in-depth training should include hands-on experience operating the advanced features in a real driving environment, which has been found to be crucial for understanding and trusting the technology (Li, Blythe, Guo, & Namdeo, 2019). Furthermore, the user interface design for new ADAS or future vehicle automation systems deserves further attention, especially with considerations of seniors’ preferences and limitations (Guo, Blythe, Edwards, Pavkova, & Brennan, 2015).

This study had some limitations in the data collection and data analysis methods. Like all focus group studies, the findings of the current study have limited generalizability to a larger population, and there was no way to rule out alternative explanations of findings, such as participants behaving in ways to meet experimenter expectations. In addition, the implementations of the ADAS differed across four car models, so the participants did not have an identical ADAS experience. Nevertheless, the focus group discussion did not reveal any model-specific issues but rather common limitations of some ADAS technology (e.g., false LKA alerts). As to the analysis method, the results produced by STM, which are the keywords identified in each topic, still need domain expertise to interpret and assign a meaningful label. In addition, STM may omit important but infrequently occurring details in the discussion, such as a unique response from one participant who may yield major design insights.

## Conclusions

This study used focus groups to examine senior drivers’ attitudes toward ADAS based on their real-world driving experience with these systems. The findings indicate that safety is seniors’ main ADAS-related consideration. To promote ADAS adoption, user interfaces should be designed to accommodate seniors’ preferences and limitations, which would typically also accommodate most nonseniors. In-depth training programs would be helpful for senior drivers in learning proper ADAS operations as well as in promoting the crucial understanding of system limitations or constraints. The findings of this study should encourage car manufacturers and policy makers to direct their efforts in vehicle design and training to aid seniors in adopting ADAS so as to enhance their mobility and safety.

## Supplementary Material

igaa017_suppl_Supplementary_Material
