# Supplementary material for: Examining Senior Drivers’ Attitudes Toward Advanced Driver Assistance Systems After Naturalistic Exposure
Source: Innov Aging. 2020 Jun 18;4(3):igaa017. doi: 10.1093/geroni/igaa017 (PMC7302428; doi:10.1093/geroni/igaa017)
Supplement: igaa017_suppl_Supplementary_Material [file igaa017_suppl_supplementary_material.pdf]

Supplementary Material

Table A1. ADAS Settings Examined in Four Vehicle Models

	**2017 Audi Q7**	**2016 Mercedes E350**	**2016 Volvo XC90**	**2015 Infiniti Q90**
**BSA**	Default setting is on;<br><br>Driver can adjust brightness.	Default setting is on;<br><br>Visual alerts displayed on the side mirror.	Default setting is on;<br><br>Visual alerts displayed on the side mirror.	Default is on;<br><br>Driver can adjust brightness.
**LA**	Driver-activated (40 mph+);<br><br>Visual and haptic alerts.	Default setting is on (37+ mph);<br><br>Visual and audio and haptic alerts.	Default setting is on (30+mph);<br><br>Audio and haptic alerts.	Default setting on;<br><br>Audio and haptic alerts.
**LKA**	Driver-activated (40 mph+);<br><br>Visual and haptic alerts.	Default setting is on (37+ mph);<br><br>Visual and audio and haptic alerts.	Default setting is on (30+ mph).	Driver-activated;<br><br>Visual and haptic alerts;<br><br>Driver can select level of intervention.
**ACC**	Default setting is on when cruise control is enabled;<br><br>Driver cannot<br><br>Disable if cruise control is enabled.	Default setting is on when cruise control is enabled;<br><br>Operates at low speeds.	Default setting is on when cruise control is enabled (can disable);<br><br>Operates at low speeds.	Default setting is on when cruise control is enabled;<br><br>Driver can disable using manual controls.

Table A2. Manufacturer Nomenclatures for the ADAS

	2017 Audi Q7	2016 Mercedes E350	2016 Volvo XC90	2015 Infiniti Q90
**BSA**	Audi side assist	Blind spot assist	Blind spot information	Blind spot warning
**LA & LKA**	Lane departure warning/ Audi active lane assist	Active lane keeping assist	Lane keeping aid	Lane departure prevention / Lane departure warning/ Active lane control
**ACC**	Adaptive cruise control	Distronic plus	Adaptive cruise control	Intelligent cruise control
